# The Glycerophosphoinositols: From Lipid Metabolites to Modulators of T-Cell Signaling

**DOI:** 10.3389/fimmu.2013.00213

**Published:** 2013-07-29

**Authors:** Laura Patrussi, Stefania Mariggiò, Daniela Corda, Cosima T. Baldari

**Affiliations:** ^1^Department of Life Sciences, University of SienaSiena Italy; ^2^Institute of Protein Biochemistry, National Research CouncilNaples, Italy

**Keywords:** glycerophosphoinositol, T-cell chemotaxis, CXCL12, Lck

## Abstract

Glycerophosphoinositols (GPIs) are bioactive, diffusible phosphoinositide metabolites of phospholipase A_2_ that act both intracellularly and in a paracrine fashion following their uptake by specific transporters. The most representative compound, glycerophosphoinositol (GroPIns), is a ubiquitous component of eukaryotic cells that participates in central processes, including cell proliferation and survival. Moreover, glycerophosphoinositol 4-phosphate (GroPIns4*P*) controls actin dynamics in several cell systems by regulating Rho GTPases. Recently, immune cells have emerged as targets of the biological activities of the GPIs. We have shown that exogenous GroPIns4*P* enhances CXCL12-induced T-cell chemotaxis through activation of the kinase Lck in a cAMP/PKA-dependent manner. While highlighting the potential of GroPIns4*P* as an immunomodulator, this finding raises questions on the role of endogenously produced GroPIns4*P* as well as of other GPIs in the regulation of the adaptive immune responses under homeostatic and pathological settings. Here we will summarize our current understanding of the biological activities of the GPIs, with a focus on lymphocytes, highlighting open questions and potential developments in this promising new area.

The glycerophosphoinositols (GPIs) are ubiquitous water-soluble phosphoinositide metabolites produced by all eukaryotic cells (1–3). Not surprisingly considering their central role in the orchestration of signaling cascades, among the phosphoinositides it is the inositol phosphates that have monopolized the scene. Accumulating evidence has however highlighted a role for the GPIs as modulators of important biological functions in a number of cell types, including T-lymphocytes, in both physiological and pathological settings. Here we will summarize our current understanding of the metabolic pathways that regulate GPI production and discuss their biological activities, focusing on T-cells.

## Biosynthesis, Transport and Degradation of GPIs

**Figure 1:**
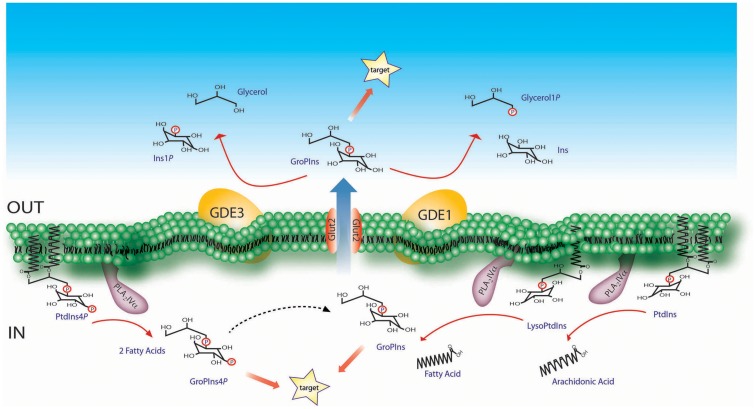
**Schematic representation of the GroPIns metabolism**. The formation of GroPIns occurs from membrane phosphatidylinositol (PtdIns) via two sequential steps, both of which are catalyzed by PLA_2_IVα. The first deacylation produces lysophosphatidylinositol (LysoPtdIns) and free arachidonic acid, since PLA_2_IVα selectively hydrolyzes phosphoinositides substituted in the *sn-2* position with arachidonic acid (49). The second deacylation releases free fatty acid and GroPIns. As indicated, PLA_2_IVα supports both of these deacylation steps, as demonstrated in *in vitro* investigations using purified phosphoinositide and lysophosphatidylinositol substrates together with the recombinant enzyme (4). Once produced in the cytoplasm, GroPIns can be active on intracellular targets or can be released through the Glut2 transporter into the extracellular space, where it can act as a paracrine factor on nearby target cells. The subsequent catabolism of GroPIns is instead located on the extracellular side of the plasma-membrane and is mediated by the GDEs. GroPIns4*P* formation, also schematized, occurs starting from membrane phosphatidylinositol 4-phosphate which is hydrolyzed, as for GroPIns, by PLA_2_IVα (unpublished observations, see main text for details).

The GPIs, which include glycerophosphoinositol (GroPIns) and its phosphorylated derivatives glycerophosphoinositol 4-phosphate (GroPIns4*P*) and glycerophosphoinositol 4,5-bisphosphate (GroPIns4,5*P*_2_), are generated from membrane phosphoinositides through two sequential deacylation reactions that are carried out by a phospholipase A_2_ (PLA_2_) and a lysophospholipase (2). Studies on the most abundant of these metabolites, GroPIns, have provided evidence that both of these reactions can be catalyzed by the same enzyme, which has been identified in thyroid cells and macrophages as the α isoform of group IV PLA_2_ (PLA_2_IVα) [(4, 5); Figure 1]. We have recently shown that the same enzymatic pathway is responsible for GroPIns4*P* production in macrophages upon treatment with a pro-inflammatory stimulus (unpublished results; Figure 1).

Glycerophosphoinositols can interact with intracellular targets and/or be released into the extracellular medium through specific membrane transporters, following their chemical gradient. The GroPIns transporter, which is responsible for the bidirectional transfer of GroPIns, was initially identified in yeast (6), and its human ortholog is the Glut2 permease (7). Reasonably, Glut2 represents only one of the mammalian GroPIns transporters, as it has cell-specific expression patterns, while GroPIns membrane permeation appears to be a general process. There is also evidence of GroPIns4*P* membrane transport. Although no specific transporter for GroPIns4*P* has been identified to date, several GroPIns4*P*-mediated activities show biochemical features that indicate specific, transporter-mediated mechanisms (8).

The half-life of GPIs is relatively short both inside the cell and in the extracellular milieu. This applies in particular to the phosphorylated, biologically active derivative, GroPIns4*P* which is rapidly metabolized within the cell, undergoing dephosphorylation to GroPIns through a Ca^2+^-dependent and GroPIns4*P*-selective activity associated with the cell membrane fraction (Figure 1). Alternatively, a Ca^2+^-insensitive activity leads to the phosphorylation of GroPIns4*P* to GroPIns4,5*P*_2_ (8). GroPIns can be reacylated to phosphatidylinositol (PtdIns) both in whole cells and in membrane fractions. At variance, no detectable reacylation of GroPIns4*P* has been documented to date (8).

The glycerophosphodiesterases GDE1 and GDE3, both of which are membrane-bound ectoenzymes, catalyze the hydrolysis of extracellular GroPIns (9, 10). GDE1 is ubiquitously expressed and hydrolyzes GroPIns to produce inositol and glycerol phosphate (9). GDE1 activity is regulated by G-protein-coupled receptors, and it is stimulated by β-adrenergic receptor agonists but inhibited by α-adrenergic receptor agonists and lysophosphatidic acid, thus providing a further level of modulation of GroPIns metabolism (9). GDE3 is a marker of osteoblast differentiation, and is predominantly expressed in mature osteocytes (11). GDE3 hydrolyzes GroPIns with a different type of attack of the phosphodiester bond, which produces inositol phosphate and glycerol (10). GroPIns4*P* is not substrate of GDE1 or GDE3, but it can compete with GroPIns for its hydrolysis by these glycerophosphodiesterases (9, 10).

## GPI Production in Immune Cells

### GroPIns is produced by macrophages in response to pro-inflammatory stimuli

A number of pharmacological and pro-inflammatory stimuli have been shown to trigger phosphoinositide hydrolysis in macrophages (2). Similar to other cell types, GroPIns production in these cells is regulated by a Ca^2+^-dependent pathway involving the PLA_2_-catalyzed deacylation of PtdIns (2). Studies on macrophages treated with cholera or pertussis toxin provided evidence that PLA_2_ is activated downstream of G proteins, catalyzing the hydrolysis of PtdIns and leading to the production of arachidonic acid derivatives and GroPIns (12). A similar pathway was identified in Kupffer cells, the resident macrophages of the liver, following stimulation with inflammatory mediators produced upon bacterial endotoxin challenge (13, 14). A concerted activation of the arachidonate pathway and production of GroPIns has been reported in several other cell types (2).

PLA_2_IVα, which had been identified as the specific, Ca^2+^-dependent PLA_2_ responsible for GroPIns production in thyroid cells (4), has been recently demonstrated to carry out this function also in macrophages. Zizza and colleagues (5) showed that PLA_2_IVα, which is abundantly expressed in macrophages, is phosphorylated by the MAP kinases Erk1/2 and by the stress-activated kinases p38 and JNK and translocates to the membrane of nascent phagosomes during Fc-Receptor (FcR)-mediated phagocytosis. A selective PLA_2_IVα activation was observed to also occur upon LPS treatment which, similar to FcR engagement, triggers arachidonic acid release (5). Moreover, pharmacological inhibition of PLA_2_IVα completely abolished both LPS- and phagocytosis-mediated GroPIns production. Interestingly, a time course analysis of GroPIns production during FcR-mediated phagocytosis revealed a persistent increase in the levels of intracellular GroPIns over time, which was paralleled by GroPIns release into the extracellular medium (our unpublished observations). This suggests that GroPIns may participate in the inflammatory responses of macrophages by acting not only in an autocrine manner, but also as a paracrine factor.

The intracellular levels of the GPIs have also been measured in T-cells. Mass spectrometry data showed that Jurkat T-cells are among the cell lines with low intracellular levels of GroPIns (45 ± 1 μM) (15, 16). Moreover, these basal levels are not increased by known pharmacological activators of PLA_2_IVα, such as Ca^2+^ ionophores, or by chemotactic stimuli, such as CXCL12, which suggests that a Ca^2+^-independent enzyme is involved in GroPIns production in these cells. Alternatively, the concentrations of arachidonoyl-substituted PtdIns, the GPI precursor, may not be sufficient to produce significant increases in the levels of intracellular GroPIns.

### Modulation of GroPIns production during immune cell differentiation

Phospholipase A_2_ activation is not only triggered by plasma-membrane receptors but also occurs during cell differentiation (17–19). Mountford and colleagues provided evidence that the levels of phosphoinositides change during the differentiation of both myeloid and lymphoid cells (20–22). Using HL60 promyelocytic cells, which can differentiate either to neutrophils in response to all-trans retinoic acid and granulocyte-colony-stimulating factor or to monocytes in response to 1α-25-dihydroxyvitamin D_3_, they showed that the intracellular GroPIns levels increased in the early stages of differentiation to either lineages, eventually doubling in fully differentiated cells. Consistent with these findings, GroPIns levels increased in neutrophils that spontaneously differentiated in culture, as compared to the initial blasts (22). Similar experiments, carried out on paired cell lines representative of immature and mature states of B-lymphocytes (Ba/F3 and NSI cells) and T-lymphocytes (S49 and C8166 cells) showed substantial increases in GroPIns levels in the cells representative of the mature states (22). Although more accurate methods to quantitate GroPIns as well as more physiological differentiation conditions will be required to validate these data, the changes in the concentrations of intracellular GroPIns suggest a role for this metabolite in the regulation of both myeloid and lymphoid cell differentiation.

### GroPIns4P production by macrophages

In addition to GroPIns, its monophosphorylated derivative, GroPIns4*P*, has been detected in several cell types, including macrophages [(2); our unpublished observations)]. PLA_2_IVα, is responsible for the production of both GPIs (i.e., GroPIns and GroPIns4*P*), based on the relative availability of the respective lipid precursors (PtdIns and PtdIns4*P*, respectively) [(4, 5); our unpublished observations]. A conundrum in these studies are the technical limitations in the accurate measurement of these metabolites. For example, the relatively high levels of GroPIns make the detection of small increases more difficult, which might explain why increases in GroPIns4*P* (which generally represents<3% of the total GPIs) do not always appear to be paralleled by increases in GroPIns. The rapid metabolism of GroPIns4*P* is a further drawback for precise determinations of its levels, although this was partially overcome by performing GroPIns4*P* measurements in the presence of orthovanadate, a general phosphatase inhibitor (2), which made it possible to monitor GroPIns4*P* increases in macrophages exposed to LPS (our unpublished observation). At variance with macrophages, no detectable production of GroPIns4*P* can be observed in T-cells (16).

## The GPIs as Modulators of T-Cell Functions: Facts and Hypotheses

### GroPIns4P promotes actin polymerization in T-lymphocytes

Cortical actin rearrangements, which are regulated by the Rho family of small GTPases (23), are crucial for a number of processes that orchestrate T-lymphocyte activation and motility (24, 25). Exogenous administration of GroPIns4*P* to fibroblasts induces the formation of actin ruffles and stress fibers by modulating the activity of Rac and Rho (26, 27), suggesting a potential role for this phosphoinositide derivative in the regulation of the actin cytoskeleton in other cell types. Treatment of both Jurkat T-cells and peripheral blood lymphocytes from healthy donors with GroPIns4*P* induces indeed actin polymerization (16), suggesting that the processes involving F-actin dynamics, including redistribution of components associated with lymphocyte motility and immune synapse assembly might be modulated by GroPIns4*P*.

The ability of GroPIns4*P* to promote actin polymerization in T-cells stems, at least in part, from its ability to induce the phosphorylation of the GDP/GTP exchanger Vav (16), which controls the activation of Rac and Cdc42 in hematopoietic cells (28). These data are consistent with the finding that GroPIns4*P* triggers a signaling cascade in fibroblasts that leads to plasma-membrane translocation of Tiam1, a Rac-specific GDP/GTP exchanger in these cells (27). This activity provides a mechanistic explanation of the agonistic effects of GroPIns4*P* on the actin cytoskeleton dynamics.

The ability of GroPIns4*P* to promote Vav activation suggests the possibility that it may also modulate gene expression. Vav initiates indeed a pathway involving recruitment to active Rac of the serine/threonine kinase Pak1, which triggers the activation of p38 and JNK that directly or indirectly activate a number of transcription factors (29). Consistent with this notion, T-cell treatment with GroPIns4*P* resulted in activation of both p38 and JNK, with a similar time course as Vav (16).

### Vav activation by GroPIns4P requires Lck

**Figure 2:**
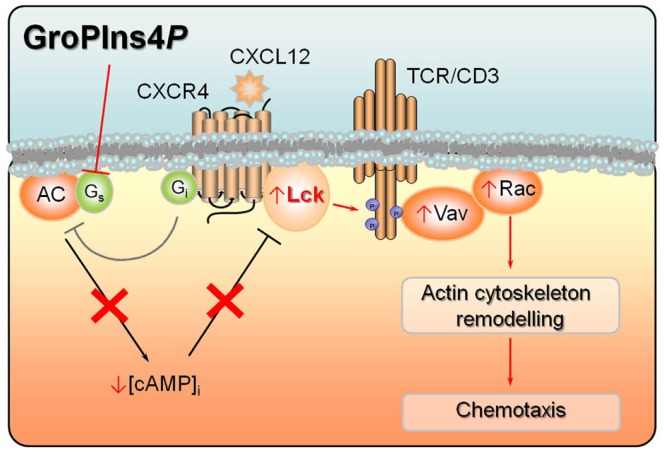
**Convergence of signals by CXCL12 and GroPIns4*P* on adenylate cyclase activity**. CXCL12 binding to its cognate receptor CXCR4 leads to activation of Lck, which stably interacts with the receptor. Lck in turn phosphorylates multiple tyrosine residues in the cytosolic tails of the TCR/CD3 complex, thereby triggering a signaling cascade involving Vav activation and eventually actin cytoskeletal rearrangements. Lck activity is further potentiated by CXCR4-dependent stimulation of Gi protein which, by inhibiting adenylate cyclase, lowers the levels of intracellular cAMP, resulting in decreased PKA-dependent activation of Csk, a negative regulator of Lck. GroPIns4*P* potentiates migratory signaling by CXCL12 by blocking the activity of Gs protein, thereby further lowering adenylate cyclase activity and hence the intracellular cAMP levels and contributing to Lck activation. Phosphorylation states and events are shown as small blue circles. Activation events are shown as arrows, inhibition events as truncated lines.

Tyrosine phosphorylation of Vav is mediated by the cooperative activity of Syk and Src family protein tyrosine kinases (PTKs) (28). We have shown that treatment of T-cells with GroPIns4*P* results in enhanced Lck activity [(16); Figure 2]. Although other mechanisms may account for Vav activation, this event is likely to be causal to the agonistic activity of GroPIns4*P* on Vav activation and the resulting actin cytoskeleton rearrangements. GroPIns4*P* fails indeed to trigger Vav phosphorylation in the Lck-deficient Jurkat T-cell variant JCaM.1 (16, 30). Moreover, GroPIns4*P* triggers Src phosphorylation in fibroblasts, which is required for plasma-membrane translocation of Tiam1 (27). Lck activation, as well as Src activation, is not a direct effect of GroPIns4*P*, at least as assessed *in vitro* (16). Unfortunately, while these studies have restricted the field, no final mechanism of action for the GPIs can be postulated, until a proteomic approach to identify direct interactors can be completed.

### GroPIns4P targets Lck-dependent signaling by modulating cAMP

In quiescent T-cells Lck is kept in an inactive state by the inhibitory kinase Csk, which becomes phosphorylated and activated by the cAMP-dependent serine/threonine protein kinase A (PKA) (31). We showed that treatment of Jurkat T-cells with GroPIns4*P* results in a decrease in the levels of cAMP, leading to impaired PKA activation and Csk phosphorylation. Hence the agonistic activity of GroPIns4*P* on Lck activation and downstream signaling results from its ability to inhibit cAMP production (16). These results are consistent with the finding that GroPIns4*P* (but not GroPIns or GroPIns4,5*P*_2_) inhibits adenylate cyclase activity in other cell types (32, 33). It is noteworthy that cAMP/PKA-mediated modulation of molecules downstream of Lck may also contribute to its effects on F-actin dynamics in T-cells (34), including Rho itself, which is phosphorylated by PKA on an inhibitory residue (35, 36).

The ability of GroPIns4*P* to activate Lck, which is responsible for initiation of the T-cell receptor (TCR) signaling cascade, strongly suggests that other signaling events, in addition to the Vav/p38/JNK pathway, might be triggered by GroPIns4*P*. One of the key targets of Lck both in TCR (37) and chemokine receptor signaling (38) is the PTK ZAP-70, which couples these receptors to multiple signaling pathways, including the Ras/MAP kinase pathway (39). GroPIns4*P* induces indeed the activation of ZAP-70 and the adaptor Shc (16), which interacts with, and becomes phosphorylated by ZAP-70 in response to TCR engagement and contributes to Ras activation by recruiting the Grb2/Sos complex (40). Consistent with its ability to promote Shc phosphorylation, GroPIns4*P* activates Erk1/2. These effects of GroPIns4*P* are crucially dependent on Lck, as they fail to occur in JCaM.1 cells (16).

### GroPIns4P enhances CXCR4 signaling

The ability of GroPIns4*P* to promote actin polymerization in T-cells profoundly influences their responses to chemokines. GroPIns4*P* (but not the other GPIs) enhances indeed CXCR4-dependent chemotaxis toward CXCL12 (16), a chemokine that regulates lymphocyte homing to secondary lymphoid organs under homeostatic conditions (39). Interestingly, the two signals may converge, at least in part, on adenylate cyclase. In fact, CXCL12-mediated activation of CXCR4 promotes the release of the α-subunit of heterotrimeric inhibitory G protein (G_i_), thereby inhibiting adenylate cyclase activity (39). Moreover, in Jurkat T-cells GroPIns4*P* reverses the cAMP-elevating activity of cholera toxin, which ADP-ribosylates the α-subunit of stimulatory G proteins (G_s_) resulting in their persistent activation. This indicates that GroPIns4*P* is able to decrease cAMP production and cAMP/PKA-dependent Csk activation by inhibiting G_s_. These mechanisms can account for the additive effect of CXCL12 and GroPIns4*P* on Lck activation [(16); Figure 2].

Recent evidence supports a crosstalk between the TCR and CXCR4. The TCR is indeed transactivated by CXCR4 which then uses the TCR machinery to elicit and potentiate downstream signaling (38, 41). Similar to other G-protein-coupled receptors, CXCR4 directly interacts with and activates Lck. The association of CXCR4 with the TCR at the cell surface allows Lck to localize in close contact with the intracellular domains of the TCR/CD3 complex and phosphorylate CD3ζ, thereby triggering downstream signaling (39). By promoting Lck activation GroPIns4*P* might potentiate the ability of CXCR4 to transactivate the TCR and hence enhance the signaling cascades leading to T-cell chemotaxis, including the pathway leading to the Lck-dependent recruitment of Shc to the CXCR4/TCR dimer.

### GroPIns4P production by bystander cells: A potential mechanism to control T-cell responses in the local microenvironment

How can the evidence obtained using exogenously added GroPIns4*P* be related to the physiological context of T-cell trafficking? Measurements of the GPI levels in Jurkat T-cells using a quantitative mass spectrometry approach revealed that these cells are among those with the lowest intracellular levels of GroPIns (15). At variance with T-cells, macrophages produce large amounts of GPIs in response to pro-inflammatory stimuli (2, 5), generating a gradient for their transporter-mediated release into the extracellular medium (2, 15, 42). We propose that these macrophage-derived GPIs may act as paracrine factors for lymphocytes. In this scenario, GPIs produced at the site of infection would enhance effector T-cell recruitment, and thereby contribute to bacterial clearance. Interestingly, among the highest GPI producers are certain tumor cells (2, 43). The release of these metabolites, combined with chemotactic signals provided by the tumor microenvironment (44), could be hypothesized to promote T-cell infiltration and activation of anti-tumor immunity.

### GPIs and T-cell fate: A working hypothesis

By promoting the activation of Lck, exogenous administration of GroPIns4*P* to T-lymphocytes triggers a Rho-family dependent pathway that is integrated with chemokine receptor signaling to potentiate T-cell chemotaxis (16). Given the central role of Lck as the initiator kinase in TCR signaling (37), it can be hypothesized that GroPIns4*P* has the ability to modulate T-cell activation. Lck has also been implicated in the regulation of T-cell apoptosis induced by a wide range of stimuli, including prolonged TCR stimulation (45) and treatment with sphingosine (46), and it is also an essential component of the signaling pathways that control Ca^2+^-mediated T-cell apoptosis, which involve both the conformational activation of Bax and the expression of proapoptotic Bcl-2 family members (47). How TCR engagement can lead to cell fates as diverse as activation, anergy, and apoptosis is one of the fundamental and as yet open questions in immunology. Investigating the effects of GroPIns4*P*, as well as of the other GPIs, on these processes, may provide valuable information on how TCR signaling is fine-tuned by these phosphoinositide metabolites to elicit different biological outcomes.

## Conclusion

The interesting scenarios opened by the studies outlined in this review using exogenously added GPIs underscore the need to address the physiological function of endogenous GPIs in the modulation of immune cell function. It has however to be underlined that the results summarized in the present review, while in part validated on normal peripheral T-cells, have been largely obtained by exogenous administration of GroPIns4*P* to Jurkat T-cells, which are known to be defective in the activity of enzymes critically involved in lipid signaling, such as PTEN (48). We have therefore to take into account a possible impact of this defect on global lipid signaling, which might also involve GPI metabolism. Since, among immune cells, the major producers of GPIs are macrophages, it will be interesting to assess the impact of this physiological source of exogenous GPIs on normal T-cells in co-culture experiments. Unfortunately the study of immune modulation by GPIs *in vivo*, which in principle could be approached using PLA_2_IVα^−/−^ mice, is prevented by lack of effect of PLA_2_IVα deficiency on GPI synthesis, at least in this model. This finding is likely to be accounted for by the fact that in PLA_2_IVα^−/−^ cells GPI synthesis is taken over by the calcium-independent PLA_2_VI, suggesting that a compensatory mechanism is activated under these conditions (15). A way to circumvent this problem in the future could be to generate an inducible knockout mouse lacking PLA_2_IVα expression in macrophages and/or T-cells, or alternatively to modulate the expression of the specific GDEs.

## Conflict of Interest Statement

The authors declare that the research was conducted in the absence of any commercial or financial relationships that could be construed as a potential conflict of interest.
